# An unusual microbiome characterises a spatially-aggressive crustose alga rapidly overgrowing shallow Caribbean reefs

**DOI:** 10.1038/s41598-020-76204-0

**Published:** 2020-11-30

**Authors:** Bryan Wilson, Chen-Ming Fan, Peter J. Edmunds

**Affiliations:** 1 Department of Zoology, University of Oxford, Oxford, UK; 2 Department of Embryology, Carnegie Institution for Science, Baltimore, MD 21218 USA; 3 Department of Biology, California State University, 18111 Nordhoff Street, Northridge, CA 91330-8303 USA

**Keywords:** Microbial ecology, Marine biology

## Abstract

Several species of crustose coralline algae (CCA) and their associated microbial biofilms play important roles in determining the settlement location of scleractinian corals on tropical reefs. In recent decades, peyssonnelid algal crusts (PAC) have become spatial dominants across large areas of shallow Caribbean reefs, where they appear to deter the recruitment of scleractinians. Our genetic investigations of PAC in St. John, US Virgin Islands, amplifying the large-subunit ribosomal RNA and *psbA* protein D1 marker genes, revealed them to be identical to *Ramicrusta textilis* previously reported overgrowing corals in Jamaica. Specimens of PAC sampled from the Honduras were likewise identical, confirming that this crustose alga inhabits the easternmost and westernmost regions of the Caribbean. We also analysed 16S rDNA tag amplicon libraries of the biofilms associated with PAC and sympatric CCA, which is favoured for coral settlement. Our results show that the microbial communities on PAC (vs. CCA) are characterized by significantly lower numbers of the epibiotic bacterial genus *Pseudoalteromonas*, which facilitates the recruitment and settlement of marine invertebrates. From these data, we infer that PAC are therefore unlikely to be attractive as settlement sites for coral larvae. Given the significant ecological change anticipated on these reefs due to increasing cover of PAC, there is an urgent need to further investigate competitive interactions between PAC and scleractinian corals, and elucidate the role of PAC and their associated microbiomes in accentuating phase shifts from coral to algae on tropical reefs.

## Introduction

As tropical reefs become increasingly degraded, many are transitioning from spatial dominance by stony corals to occupation by macroalgae^1^. Through competition with macroalgae for space on hard substrata, the distribution and abundance of coral recruits is restricted, as is their post-settlement success, thus favouring profound changes in coral reef community structure^2–4^. Since the 1980s, Caribbean reefs have been exposed to multiple agents driving large-scale declines in coral cover, including diseases causing mass mortality of the echinoid *Diadema antillarum*^5^ and the coral *Acropora* spp.^6^, seawater warming^7^ leading to global coral bleaching in 1987, 1998, 2005, and 2016^8–11^, and major hurricanes^12^. In a region (i.e., the Caribbean) classically characterised by low rates of coral larval recruitment (i.e., circa 100 corals m^−2^)^13–15^, compared to the Indo-Pacific^16,17^, and challenged by overfishing^18,19^, many present day reefs have undergone changes in benthic community structure to favour taxa that grow faster than scleractinian corals. These include macroalgae^4,20^, sponges^21^, and octocorals^22^ to the detriment of scleractinian coral cover.

Recruitment of pelagic larvae to benthic surfaces by scleractinians is one of a number of processes necessary for populations of scleractinians to recover following severe disturbances^14^. Freshly released brooded coral larvae, as well as those developing from mass-spawned gametes, rely upon a complex sequence of habitat-specific cues at increasingly smaller scales^23^ to select a suitable settlement location, and then initiate attachment to, and metamorphosis on, this surface^24,25^. Several decisive factors are involved in settlement choices by coral larvae, the best documented of which are the physical properties of the substratum (e.g., rugosity and light microenvironment) and the presence of select species of crustose coralline algae (CCA)^26,27^ and their associated microbial biofilms^3,28–32^. Within these microbial biofilms, members of the bacterial genus *Pseudoalteromonas*, that have been isolated from tissues of CCA (including *Hydrolithon onkodes* and *Neogoniolithon fosliei* from the Great Barrier Reef and *Paragoniolithon solubile* from the Caribbean), are potent inducers of settlement and metamorphosis of coral larvae^24,33,34^. In these cases, larval settlement is induced by the secondary metabolite tetrabromopyrrole (TBP) produced by the bacteria^34,35^. Recent work^36^ has also reaffirmed the earlier view that certain CCA can directly promote coral settlement without a participatory role of bacteria^37,38^.

**Figure 1:**
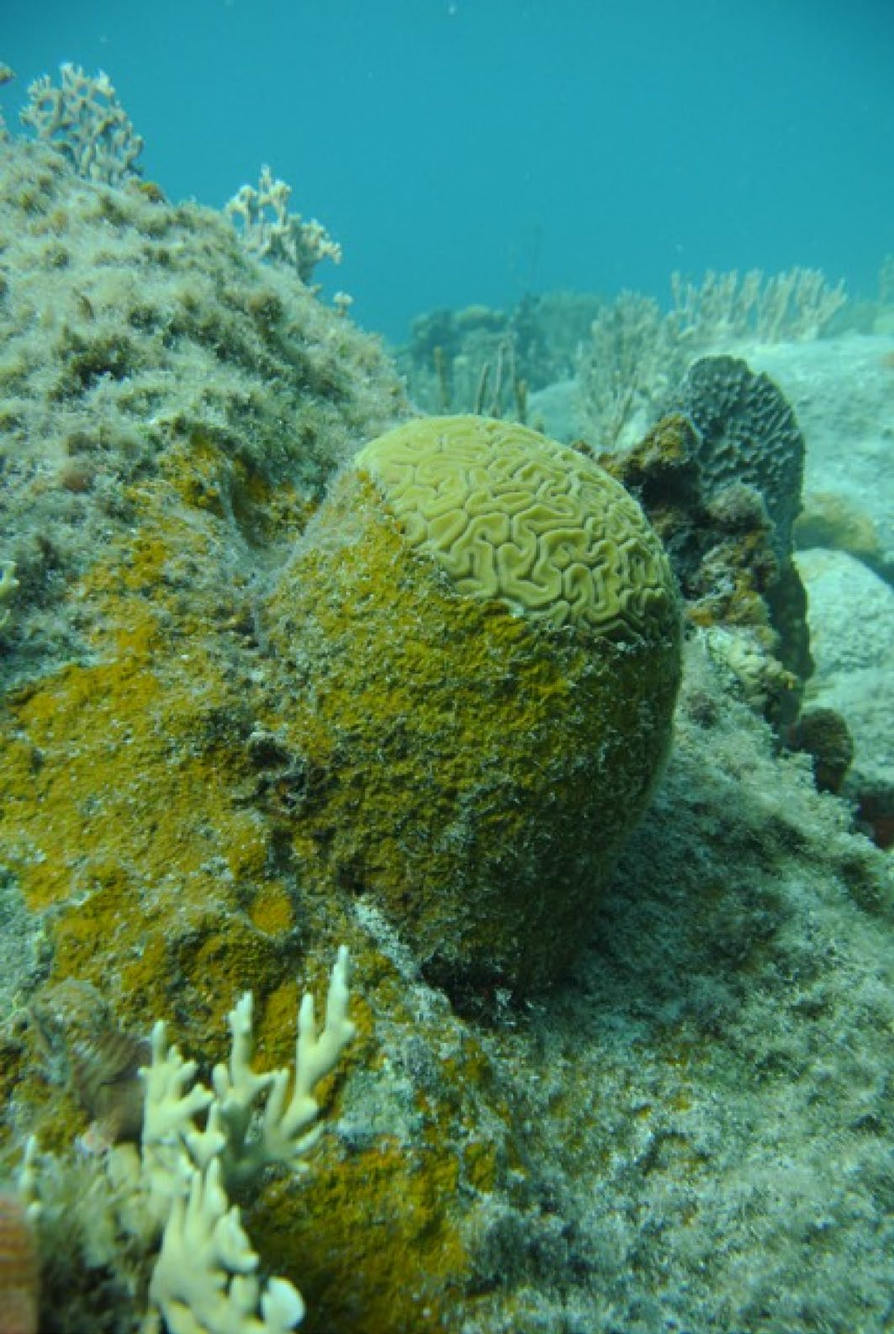
PAC overgrowing *Diploria labyrinthiformis* on reefs of St. John, US Virgin Islands.

In 2010, a new threat to Caribbean reefs emerged as aggressively spreading peyssonnelid algal crusts (PAC), which were first documented in Florida and Belize^39,40^ and later in Jamaica^41^, Bonaire, Netherlands Antilles^42,43^, Puerto Rico^44^ and now in St. John, US Virgin Islands^45^. PAC have been reported as common on shallow coral reefs ($<3$ m depth) in these locations, and in Bonaire for example (Lac Bay in 2010), PAC aggressively grew over vacant space, corals (e.g., example from St. John, Fig. 1), and sponges to occupy as much as 18.7 ± 20.9% of the benthos^43^. Rhodophyte crusts, including CCA and PAC, are common on shallow continental shelves throughout the world^46^, and while peyssonnelids have long been components of the algal flora of Caribbean reefs^47–50^, their recent appearance as spatially dominant algae on shallow reefs is novel for these locations^41^. It is likely that PAC are a biodiverse group composed of endemics, for example, *Peyssonnelia stoechas* in St. John, US Virgin Islands (R. Steneck, unpublished data), *Metapeyssonnelia milleporoides* in Puerto Rico^44^, and *Ramicrusta* sp. from Bonaire and Puerto Rico^51,52^. The genus *Ramicrusta* was established in 1981, based on samples described from the Paracel Islands, South China Sea^53^, and prior to its detection in Jamaica^41^, it was unknown in the Caribbean. Since 2013, PAC have encroached upon large areas of space on some shallow reefs ($< 7$ m depth) in St. John^54^, with surveys at 3 m depth showing an increase in mean cover of PAC from 8.5 ± 3.6% (mean ± SE, n = 5 sites) in 2015^54^ to 22.0 ± 9.1% (mean ± SE, n = 5 sites) in August 2017^45^, with up to 34.5 ± 3.2% (mean ± SE, n = 39) cover at an exposed headland. Two months later, high cover of PAC persisted after two Category Five hurricanes impacted St. John in September 2017^45^. To date, 14 weeks of fieldwork over 3 years in St. John have revealed only two coral recruits on PAC, and rare signs of fish herbivory on these crusts. The reasons underlying the novel ecological success of PAC on present-day Caribbean reefs remain unknown.

Crusts of CCA (family Corallinaceae, Rhodophyta) can promote coral recruitment through microbial flora associated with their surface^33–35,55^. As PAC have been reported to deter larval settlement and metamorphosis^45,56^, and different species of macroalgae harbour unique and species-specific bacterial communities^32,57^, here we asked whether PAC might harbour microbial consortia differing from those associated with CCA. We compared microbial communities in surface mucous (SM) from PAC (collected from igneous rocks) and also from CCA (on both igneous rocks and carbonate substrata) sampled from shallow reefs of St. John. Using high-throughput sequencing (HTS), we analysed amplicon libraries of biofilms associated with each algal crust. Using additional molecular and microscopic tools, we identified the algal species comprising PAC aggressively overgrowing shallow reefs in St. John.

**Figure 2:**
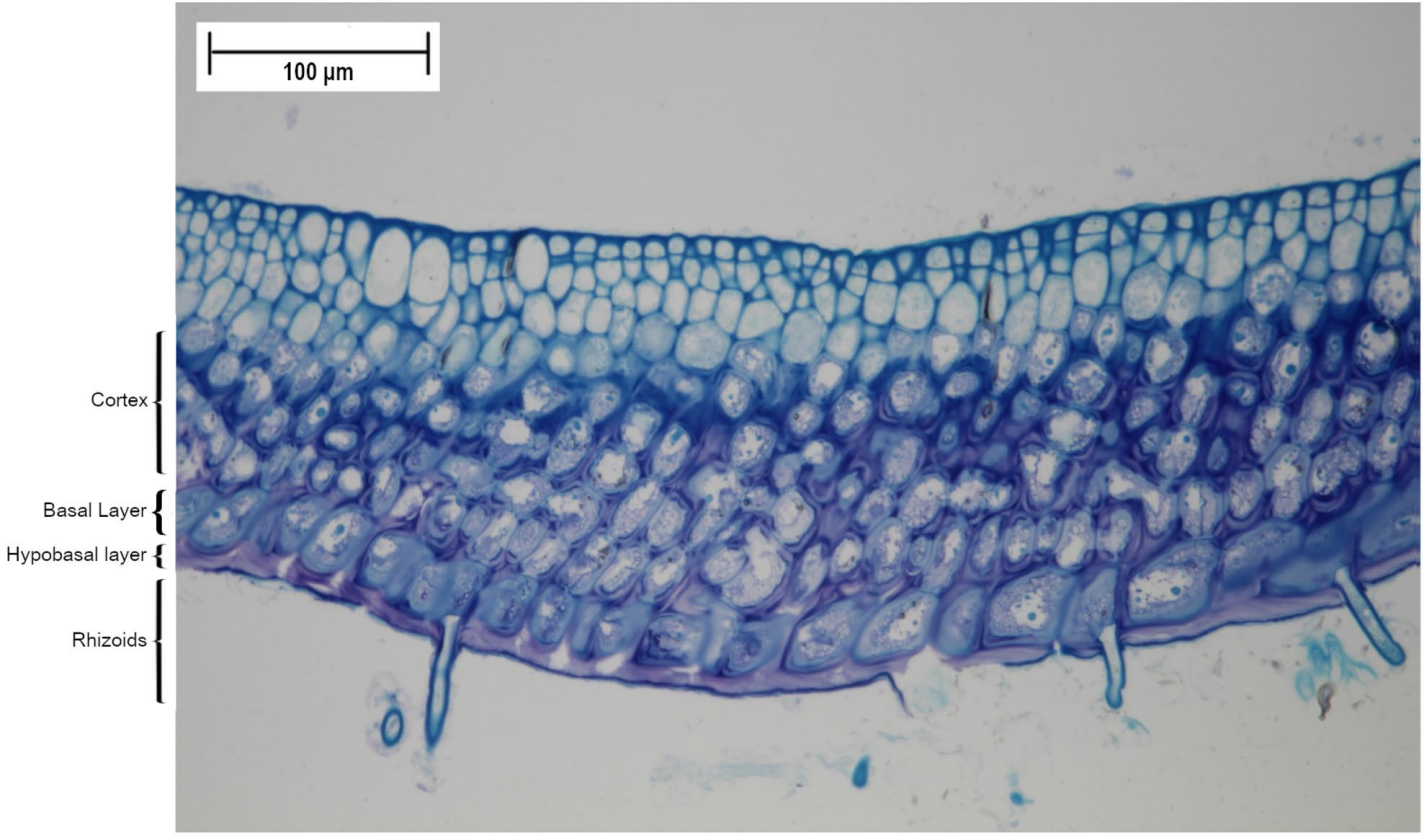
Light microscopy of decalcified *Ramicrusta textilis*. Radial vertical section (at 20$\times$ magnification) showing basal layer and rhizoids.

## Results

The samples of PAC removed from the reefs of St. John in August 2018 supported a definitive identification of the alga involved. BLASTn comparision with the NCBI database for both *LSU* (590bp) and *psbA* (410bp) gene fragments amplified from PAC genomic DNA, resulted in 100% sequence identity with voucher specimens of *Ramicrusta textilis* (Accession Numbers FJ848970 and KM360015, respectively) sampled from the coastal waters of Jamaica in 2003^41^. Low resolution ($\times$ 20) microscopy of radial vertical sections of the PAC (Fig. 2), showing the well-defined tabular layer of basal cells and abundant rhizoids on the alga’s lower surface, also confirmed that the specimens were identical to those previously described by Pueschel and Saunders^41^.

**Figure 3:**
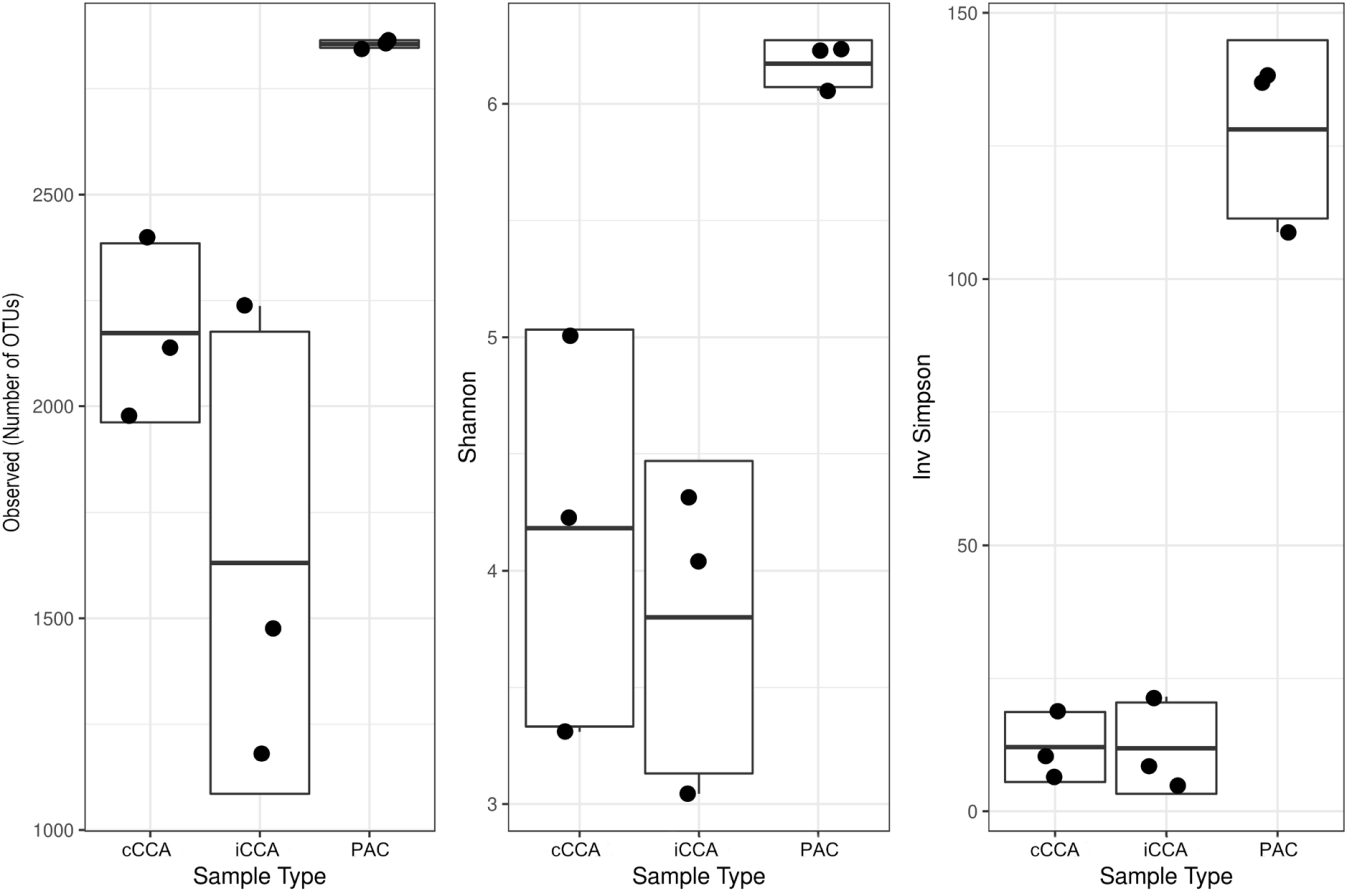
Alpha Diversity indices (Observed, Shannon and Inverse Shannon) of CCA and PAC SM prokaryote richness and diversity (based on OTUs derived from HTS of 16S rDNA amplicons). The ends of the box represent the 25th and 75th percentiles, the whiskers represent minimum and maximum range, the band inside each box represents the median and the black filled circles represent the samples. iCCA = igneous CCA (n = 3), cCCA = carbonate CCA (n = 3), PAC = peyssonnelid algal crusts (n = 3).

The microbiome diversity (in terms of operational taxonomic units [OTUs], defined here by unique 16S prokaryote ribosomal RNA gene sequences) associated with PAC differed from that of CCA, with richness (total number of observed OTUs) and diversity (Shannon and Inverse Simpson) significantly higher in microbes associated with PAC (Fig. 3). Of the 7955 OTUs recovered, 73.6% were in at least one sample from PAC, compared with 47.7% and 59.6% in samples from carbonate-associated CCA (hereafter cCCA) and igneous-associated CCA (hereafter iCCA), respectively. The numbers of unique OTUs in each sample type were similarly proportioned, with 23.1% associated with PAC, 3.0% with cCCA, and 5.8% with iCCA. There was a small core microbiome (sensu^58^) consisting of 203 OTUs (2.6%) in samples regardless of source, whilst within samples from algal crusts, the core microbiome from PAC (1705 OTUs) was more diverse than from cCCA (583 OTUs) or iCCA (941 OTUs). A one-way Analysis of Similarity (ANOSIM) revealed a significant difference in OTU community structure between the two crustose algal types (Global R = 1.0, $p \le$ 0.01); however, ANOSIM of the CCA samples only, showed that there was no significant difference between microbial communities associated with CCA on the two different substrate types (Global R = 0.148, $p \le$ 0.3). Prokaryote diversity in samples from PAC was greater than in samples from CCA, and yet the diversity between samples from PAC was more conserved than in CCA (Fig. 3).

**Figure 4:**
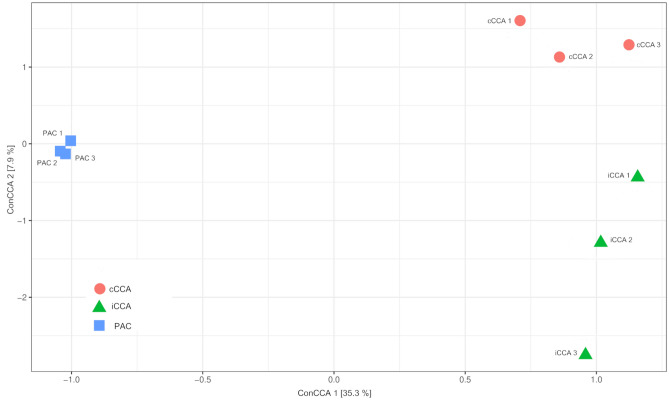
Constrained Canonical Analysis of prokaryote community structure (based on OTUs derived from HTS of 16S rDNA amplicons) by crustose alga type. iCCA = igneous CCA (n = 3), cCCA = carbonate CCA (n = 3), PAC = peyssonnelid algal crusts (n = 3).

**Figure 5:**
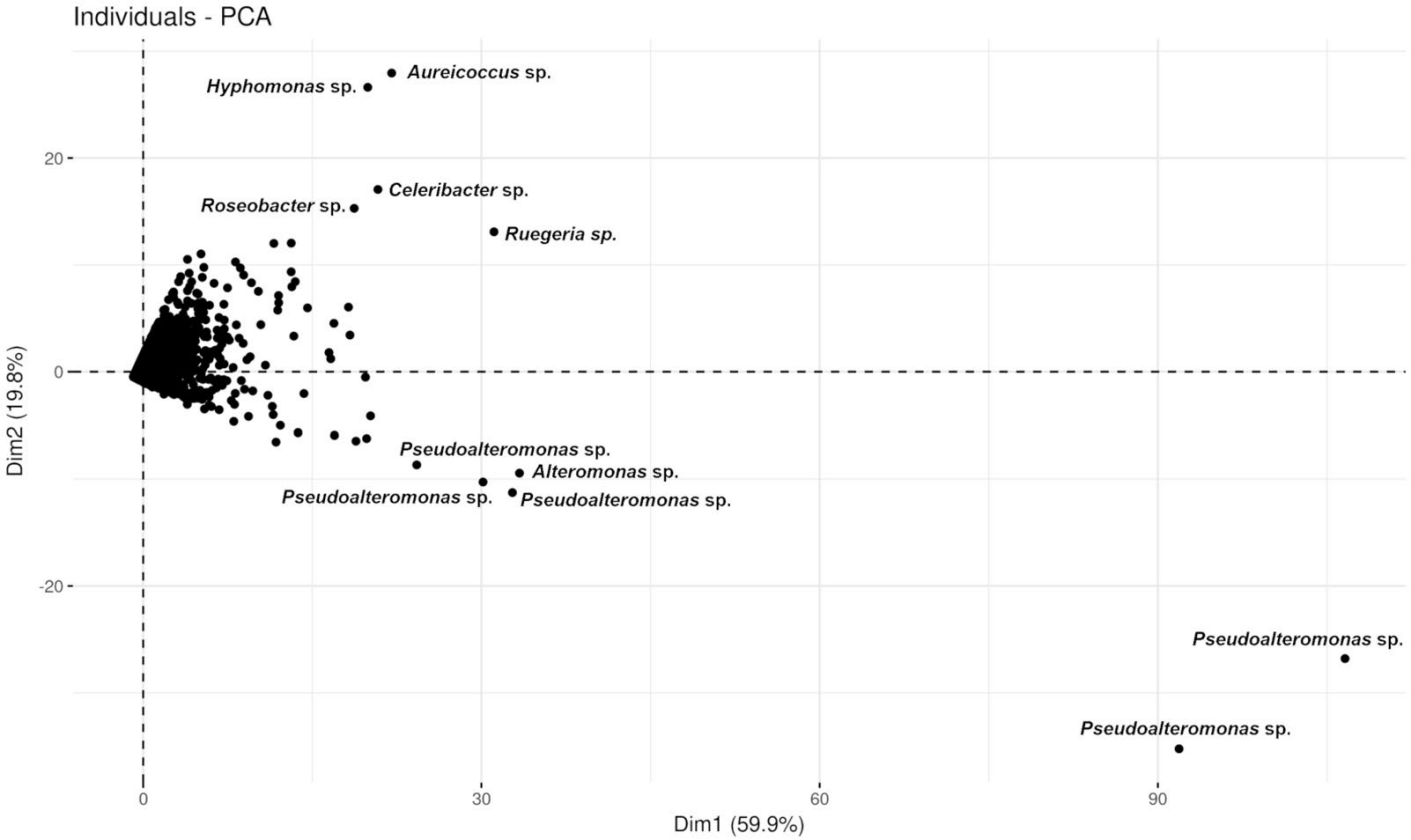
Two-dimensional principle component analyses (PCA) showing the variance in communities of prokaryote OTUs associated with CCA and PAC SM microbiomes.

Constrained canonical analysis (ConCA) of the OTU data (Fig. 4) confirmed that the microbial communities on PAC tightly clustered on the first axis (ConCA1), and were distinct from the more divergent microbial samples from CCA. Microbial communities from cCCA and iCCA separated similarly from PAC but on the second axis (ConCA2), indicating that crustose algal type strongly influenced the prokaryote community, and that communities associated with CCA on different substrata were more similar to each other than to PAC. The weighting of individual OTUs in describing variation among samples was investigated by Principal Component Analysis (PCA), with a scatterplot of square-root transformed PCA data for OTUs highlighting the predominant contribution of a group of OTUs along the first axis (Dim1) comprising almost entirely of sequences affiliated with *Pseudoalteromonas* spp. (Fig. 5).

**Figure 6:**
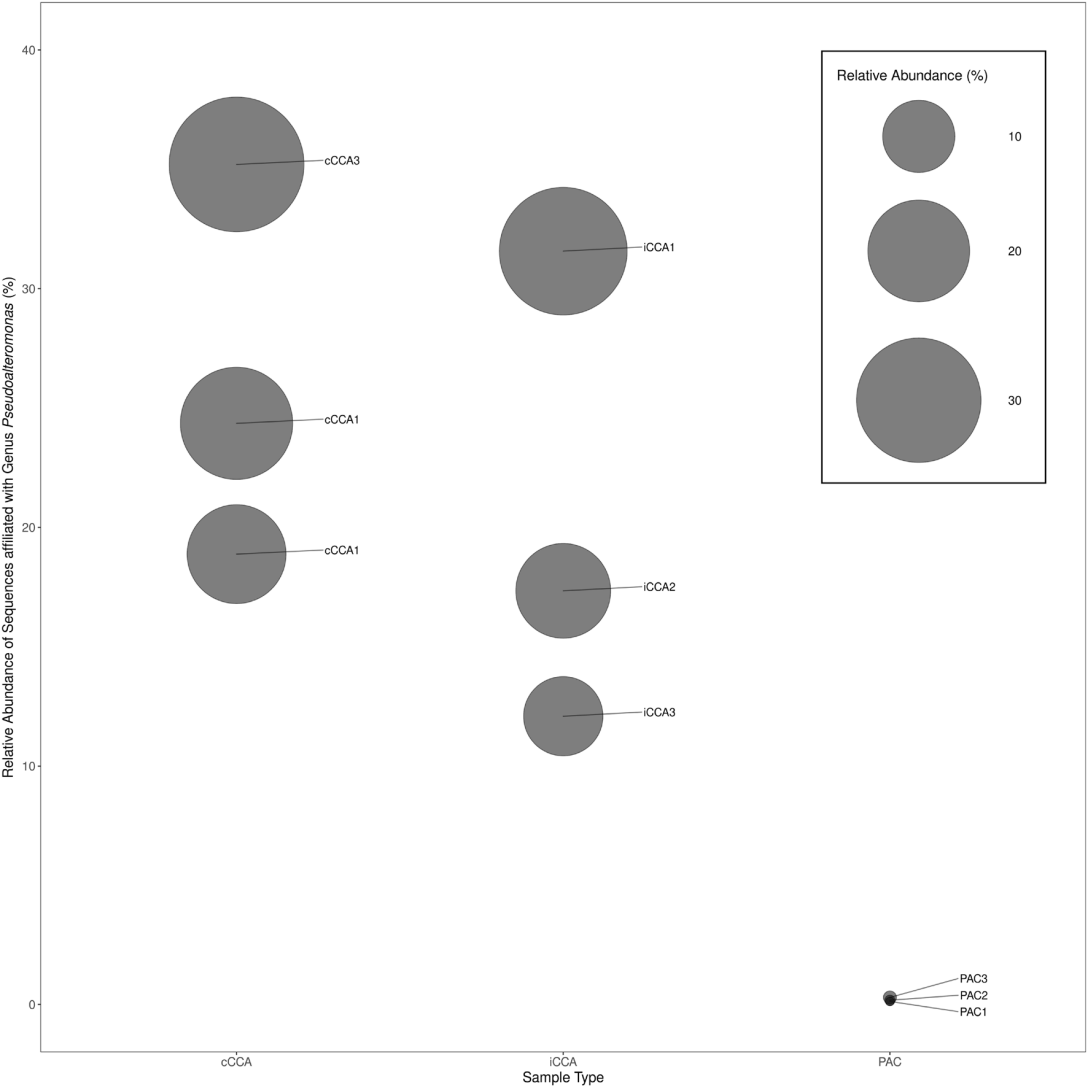
Relative abundance of the genus *Pseudoalteromonas* in prokaryote communities associated with CCA and PAC SM. iCCA = igneous CCA (n = 3), cCCA = carbonate CCA (n = 3), PAC = peyssonnelid algal crusts (n = 3).

The relative abundance of *Pseudoalteromonas* spp. OTUs from algal crusts, revealed a large difference between samples harvested from PAC and CCA (Fig. 6). Sequences related to *Pseudoalteromonas* spp. were present at low relative abundance (0.14 ± 0.04% of reads [n = 307,225 ± 15,415]) in samples (n = 3) from PAC, but had over a hundredfold greater abundances (21.33 ± 8.63% of OTUs [n = 131,100 ± 14,824]) in samples (n = 6) from CCA.

## Discussion

Surveys of shallow reefs ($\le$ 14 m depth) along 5 km of the south shore of St. John revealed that rocks coated in CCA and algal turf are common settlement substrata for scleractinian recruits (reported in more detail in^45^). Densities of small scleractinians ($\le$ 4 cm diameter) routinely are circa 12 colonies m$^{-2}$ on these surfaces^59^, yet only two small scleractinians that putatively settled on PAC were found during the surveys conducted for densities of small corals over 2017–2019. Although the abundance of PAC in July 2017 varied strongly among quadrats, some of the quadrats were 83% occupied by PAC. Thus, it is reasonable to infer that scleractinian larvae were repelled from settling on benthic surfaces dominated by PAC, or alternatively, that they settled but were overgrown or subsequently removed by grazing or detachment. Our study provides the first evidence that contemporary sheets of PAC in the Caribbean harbour very low population densities of bacterial species previously implicated in promoting coral larval recruitment. Using HTS of 16S rDNA amplicons derived from the SM of two types of crustose algae, sequences affiliated with *Pseudoalteromonas* spp. bacteria were found in significantly lower numbers on PAC versus CCA, where they dominated the microbial consortia (Fig. 6). Given the dramatic and recent increase in abundance of PAC on shallow reefs in the Caribbean^39–44^, the absence of scleractinians recruiting to its surfaces^45^, and the strong role of *Pseudoalteromonas* spp. in the settlement of some marine invertebrate larvae^31^, we hypothesise that the paucity of this microbial genus on PAC may contribute to the recent ecological success of this functional group of algae in the Caribbean.

The initial impetus for this present study was to follow-up on previous research^60^, which investigated the factors influencing coral recruitment to, and post-settlement success on, different types of rock (i.e. igneous versus carbonate) on the reefs of St. John. This previous work suggested that larvae did not discriminate between rock types at settlement^60^. The present study was conducted to test the hypothesis that the microbial consortia differed between putative settlement surfaces in St. John (i.e. igneous versus carbonate rock) and, therefore, could provide a means to mediate larval settlement behaviour and distribution of juvenile corals. PAC microbiomes were concurrently sampled from the same site to provide an ecological context to the CCA, as a crustose alga which shared the substrata with CCA, but on which very few coral recruits had been observed. Whilst the OTU richness was higher in microbial communities associated with cCCA than iCCA (Fig. 3), substratum type had no significant effect on epibiotic microbial community composition; cCCA and iCCA were associated with similar relative abundances of *Pseudoalteromonas* spp. (18.1% ± 9.0 s.d and 24.6% ± 8.5 s.d, for iCCA and cCCA respectively). Interestingly, collection site (i.e. sampling locations along 4 km of the south shore of St. John) had a greater effect on CCA microbial composition than substratum type, with a PCA of prokaryote OTUS showing samples of both iCCA and cCCA clustering closely together by site, but with sites clearly separated (see Supplementary Fig. S1 online). These findings therefore complement the empirical data in the previous study^60^, by revealing that rock surfaces to which corals recruited in proportionately equal numbers were not distinguishable in terms of the microbial communities on their surfaces.

The majority of obligate marine *Pseudoalteromonas* spp. share intimate associations with diverse eukaryotic hosts^61^, where they are constituents of the microbial community occupying the biofilms coating their exposed outer surfaces. *Pseudoalteromonas* spp. are well suited to this niche, because many of its species produce anti-bacterial compounds^62^ and employ quorum sensing^63^ to aid their competitive colonisation of surfaces. Additionally, many secrete extracellular polymeric substances (EPS) essential for biofilm structural integrity^64^. However, it is their involvement in the larval metamorphosis and settlement stages of marine invertebrates^30,55,63,65,66^, and in particular with several species of CCA^24,33^, which has garnered most recent interest. The primary means by which *Pseudoalteromonas* spp. contribute to the settlement of scleractinian larvae appears to be through the production of the secondary metabolite, tetrabromopyrrole (TBP), which is a potent inducer of larval settlement and metamorphosis in several species of coral^34,35^. More recently, a second mechanism for the induction of invertebrate metamorphosis by *Pseudoalteromonas* spp. has been suggested in larvae of the tubeworm *Hydroides elegans*, where ordered arrays of phage tail-like metamorphosis-associated contractile structures (MACs), mediate bacterial-host interactions^66^. MACs in *Pseudoalteromonas luteoviolacea* induce *H. elegans* larval settlement, initiate loss of host cilia and activate host metamorphosis. As of yet, this mechanism has only been investigated in *H. elegans*, but genomic comparisons of *P. luteoviolacea* with other bacterial strains known to induce settlement in the tubeworm revealed it to be unique in producing these structures^67^, reinforcing the importance of this bacterial genus in the life cycles of various marine invertebrates.

Whilst the disproportionate numbers of sequences related to *Pseudoalteromonas* spp. on CCA and PAC provide compelling circumstantial evidence of differential capacity to induce larval settlement, the contrasts in overall microbiome diversity between the two algal types are also of great interest. The richness of prokaryotes in both the total (Fig. 3) and core microbiomes of PAC was significantly higher than those on the CCA, yet there was less variability between independently sampled microbiomes from PAC versus CCA, suggesting a selective pressure of PAC on their core microbiome. The identification of core OTUs is oftentimes important in microbial ecology, as it has been suggested that organisms that occur in all host species (or indeed habitats) are likely critical to the functioning of that particular community^58^. Metagenomic analysis of the microbiome of PAC will likely offer a greater insight into the functional roles of the core microbiome (and in particular, *Pseudoalteromonas* spp.) and the maintenance of what appears to be a stable symbiotic relationship between the PAC and their epifauna.

Present-day large scale crusts of PAC in the Caribbean, which are likely to include at least two speciose genera (*Peyssonnelia* spp. and *Ramicrusta* spp.), have not been extensively studied, and until now, little information is known regarding the taxonomic identity of the species composing the crust, their ecology, or their range distribution^43,68,69^. A recent study in which PAC were observed to be overgrowing corals in the South China Sea revealed that crusts frequently co-occurred in a complex which comprised *Ramicrusta textilis*, members of the family Peyssonneliaceae and also the genus *Lobophora*^70^. Peyssonnelid algae often are also only identified to genus, as species share a simple gross morphology (as in^69^). However, the recent increased abundance of PAC in the Caribbean^41^, has motivated augmentation of traditional morphological taxonomy with molecular taxonomy, the application of which has identified several new taxa including *Ramicrusta* sp. in Bonaire^52^. Prior to the present study, only one sample from the US Virgin Islands had been identified morphologically as *Peyssonnelia stoechas* (R. Steneck, Pers. Comm.). However, now that the PAC from one site on the south shore of St. John have been identified as *Ramicrusta textilis*, future research efforts can begin to address the critical need for a widespread comparative analysis of taxonomic composition of the PAC spreading rapidly on shallow Caribbean reefs. A full-length CDS for the *psbA* gene (Accession Number MT215153) from *Ramicrusta textilis* recently sampled on reefs in St Thomas (also in USVI) shows 100% sequence identity to the partial *psbA* gene fragments elucidated in this study (P. Gabrielsen, Pers. Comm). A preliminary survey of reefs in the Honduras in the Western Caribbean has also revealed that PAC found there also share 100% sequence identity with the specimens described in the present analysis of PAC samples from St. John (ENA Accession Number LR828131). An important conclusion supported by these results, is that *Ramicrusta*-dominated PAC may now have a cosmopolitan range in the Caribbean. A draft genome assembly is also currently in preparation by one of us (B. Wilson) which will likely increase our knowledge of PAC biology.

Whilst much of the biology of tropical PAC remains to be more completely described, and most studies which have addressed the ecology of benthic reef communities accord scant mention to PAC, a few details regarding their biology are unequivocal. First, their high abundance in shallow water on present-day Caribbean reefs is a recent development that first gained attention around 1998^39^, and the trend has been noted along a 3000-km arc extending from Honduras (B. Wilson, unpublished data) to Jamaica^41^, Puerto Rico^44^, St. John^54^, and Bonaire^42^. Most records of this new phenomenon have come from shallow reefs (i.e., $\le$ 3 m), but in St. John it is spreading to at least 14 m depth, where in July 2017, it covered a mean of 3.3 ± 0.6% (mean ± SE, range 0–13%, n = 30) of the benthos. South of St. Thomas, PAC has been found in $>75\%$ of non-overlapping video clips from 30 m depth at College Shoals East^71^. Whilst PAC have been recorded on Caribbean reefs since at least the 1920’s^47,72,73^, and across multiple habitats, its recent increase in abundance in select locations probably reflects the combined effects of multiple factors. Of these, whilst it has been suggested that *Ramicrusta* found in PAC is invasive to the Caribbean^42^, the ongoing trend for phase-shifts from coral- to macroalgal-domination of Caribbean reefs^18,74^ may have also have favoured increased abundances of endemic PAC (e.g. *Peyssonnelia* spp.) along with fleshy macroalgae.

However, one interesting aspect of its biology that might be contributing to the current ecological success of PAC in the Caribbean may be its ability to deter settlement by invertebrates upon its surface. As the present study highlights, this likely reflects the effects of a combination of potent chemical, microbial and mechanical defenses. Many marine algae are protected from herbivory by either secondary metabolites^75^, or high concentrations of calcium carbonate^76^, with different groups of fish affected by each mode of deterence^77^. *Peyssonnelia* spp. in Fiji produce novel sesquiterpene hydroquinones called peyssonneic acids^78,79^ that inhibit the growth of *Pseudoalteromonas bacteriolytica*^80^, a pathogen of the commercially important macroalga *Laminaria japonica*^81^. The exclusion of *Pseudoalteromonas* spp. by PAC may therefore potentially be a pathogen defense mechanism, an indirect result of which is the inhibition of coral larval settlement. PAC may accentuate their competitive advantage on present day reefs by employing both defensive mechanisms (secondary metabolite production and high calcification) in concert, therefore effectively repelling a broad range of grazing fish and potentially utilising the same mechanisms in their aggressive overgrowth of live corals. However, as the spread of high-cover growths of PAC throughout the Caribbean seems to be a relatively recent phenomenon, it is likely that multiple factors have contributed to its success on present day reefs.

This study highlighted the significant difference in microbial communities associated with CCA versus PAC, in particular illuminating the disparity in the relative abundances of sequences related to the ubiquitous and ecologically-important marine bacterium *Pseudoalteromonas*. Given the ongoing and severe decline in coral abundance in the Caribbean, further investigations into the role of *Pseudoalteromonas* in coral settlement and the indirect means by which its populations may be affected by PAC are necessary.

**Figure 7:**
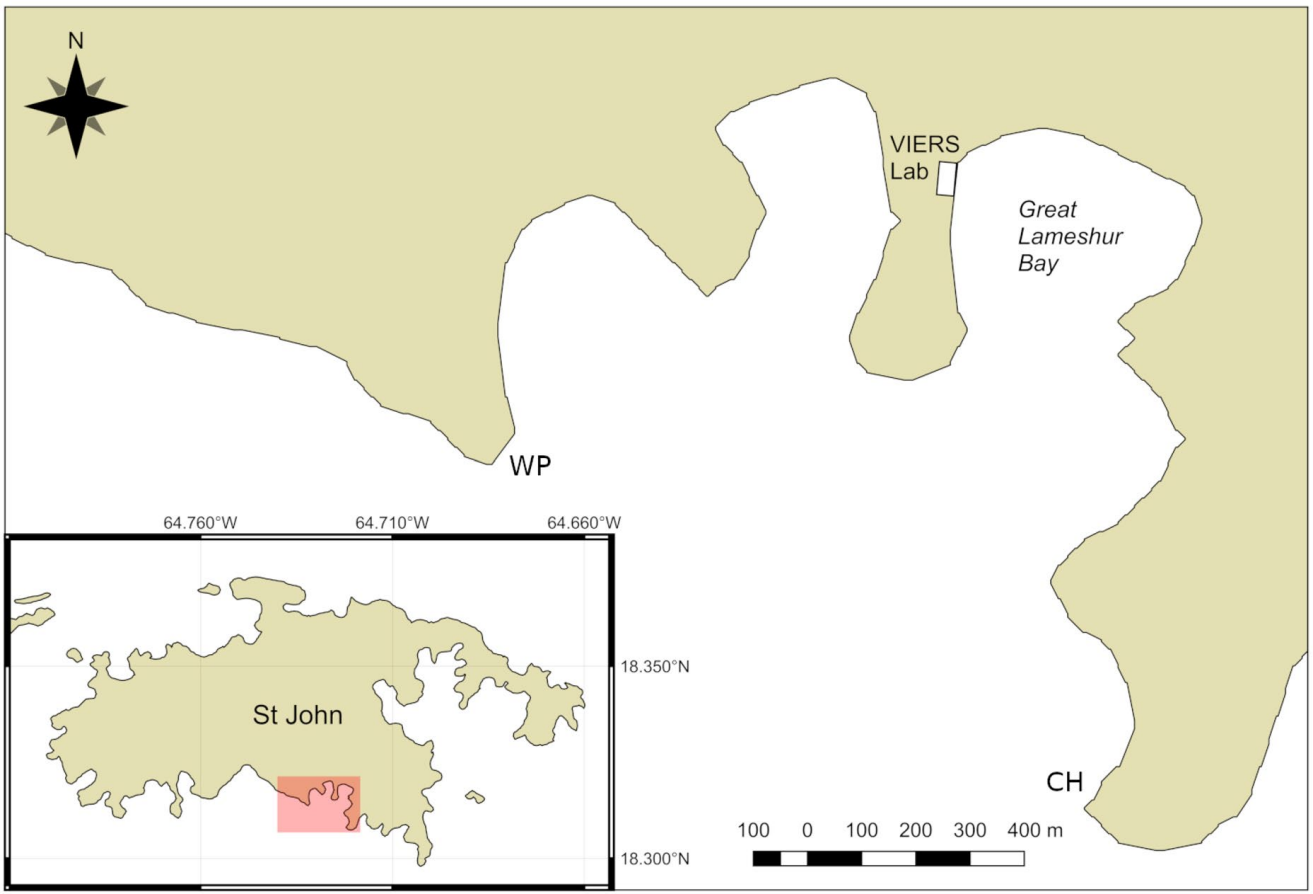
Map showing study site at Lameshur Bay, St. John, US Virgin Islands. Map created using QGIS (v2.18.17; https://qgis.org/en/site/) under GNU General Public License (Version 2)^98^, using Natural Earth vector and raster map data (http://naturalearthdata.com/). WP = White Point, CH = Cabritte Horn, VIERS = Virgin Islands Environmental Resource Station.

## Methods

### Sampling

Mucous swabs of crustose algae (PAC and CCA) were collected for microbiome studies at 7–10 m depth across Great Lameshur Bay, St. John, US Virgin Islands ($18{^{\circ }} 18^{\prime} 25.1448^{\prime \prime }$ N $64{^{\circ }} 43^{\prime } 14.4248^{\prime \prime }$ W), in August 2016 (Fig. 7). PAC was defined as a crust of peyssonnelid algae, thought to be in the genera *Peyssonnelia* and *Ramicrusta*^41,51,52,54^, with pooling of taxa into a single functional group (PAC) rationalized by their convergence in function^82^. Patches of CCA and PAC were haphazardly selected for sampling at various locations on the shallow fringing reefs between White Point (WP) and Cabritte Horn (CH) (Fig. 7) where scleractinians, octocorals sponges, and macroalgae are common, but where coral cover has remained $< 4.5\%$ for decades^59,83^. PAC were sampled from igneous substrata (n = 3), whilst CCA were sampled from both igneous (n = 3; see Supplementary Fig. S2 online) and carbonate rock (n = 3; see Supplementary Fig. S3 online) surfaces, with this sampling regime reflecting both the profusion of igneous boulders and submerged cliffs on the shallow reefs of the Virgin Islands, and the distribution of PAC and CCA on these surfaces. CCA was sampled from two rock types in order to determine whether substratum type was associated with surface mucous microbiome composition. Sterile cotton swabs were stored prior to sampling in sealed, sterile 15 mL polypropylene tubes. Inverted tubes were opened underwater immediately above the crustose algae and their SM was collected by rolling the swab over the algal surface, and returning the swabs to the inverted tube such that it was sealed with minimal seawater ingress and the swab maintained in an air pocket. Upon return to the laboratory ($< 60$ min after sampling), swabs were trimmed to 2 cm length and added to cryogenic vials (2 mL; Corning, Part No. 430488) containing 500 $\upmu \hbox {L}$ RNAlater (ThermoFisher Scientific), with the swab fully immersed. Cryogenic vials were stored at − 20 °C in St. John for five days, but were thawed to room temperature for shipment to Europe where they were stored at 4 °C prior to analysis. The collection of mucous samples in St. John was permitted by the Virgin Islands National Park (VIIS-2016-SCI-0031).

For microscopic examination and phylogenetic identification of the PAC, tissue samples were collected in August 2018 from an area of a few square metres at approximately 4 m depth at Cabritte Horn. Upon immediate return to the laboratory, tissue samples were carefully examined and brushed clean to remove as much contaminating debris (e.g. macroalgae and invertebrates) as possible. Prepared samples were immersed in RNALater and stored at 4 °C, prior to shipment at room temperature to the Department of Embryology, Carnegie Institution for Science, Baltimore, USA. The collection of tissue samples in St. John and their export was permitted by the Virgin Islands National Park (VIIS-2018-SCI-0012) and Department of Planning and Natural Resources (DFW 18087J).

### Microscopy

Specimens for microscopy were fixed for 4 h in a solution containing 3% glutaraldehyde and 1% formaldehyde in a 0.1 M sodium cacolyte buffer (containing 2 mM each calcium and magnesium salts) at pH 7.4. Samples were rinsed for 10 min (four times each) in distilled water before being decalcified, firstly in 10% formic acid at $4 {^{\circ }}\hbox {C}$ for 3 d and then in fresh 10% formic acid (at $4 {^{\circ }}\hbox {C}$ again) overnight. After two further rinses (10 min each) in distilled water, specimens were dehydrated for 15 min in each of a graded ethanol series (35%, 50%, 75% and 95%), before two final 100% ethanol treatments for 15 min. Specimens were then treated twice with a solution of 100% ethanol and resin (either LR White or Micro-Bed), for 1 h with ethanol:resin (2:1), then 1 h with ethanol:resin (1:2). Specimens were infiltrated with 100% resin overnight at $4 {^{\circ }}\hbox {C}$, before two further treatments of 100% resin for 1 h each. The final embedding in 100% resin was carried out at $55 {^{\circ }}\hbox {C}$ overnight. $1\upmu \hbox {m}$ sections were cut and mounted on slides and stained with 1% Methylene Blue (for visualisation of cell walls) and 1% Azure B (cytoplasm), warm, for light microscopy.

### PAC Microbiome DNA Extraction, PCR and HTS

Genomic DNA was extracted from mucous swabs using a modified protocol for the Fast DNA SPIN Kit for Soil (MP Biomedicals). Briefly, the contents of the Lysing Matrix E tube were transferred to the sample vial containing the cotton swab. Sodium Phosphate Buffer (300 $\upmu \hbox {L}$) was added to the sample and vortexed for 5 s. MT Buffer (122 $\upmu \hbox {L}$) was added to the cryogenic vial and the sample homogenised on a Pulsing Vortex Mixer (VWR) at maximum speed for 40 s. The sample was centrifuged at 10,000 $\times$ g for 10 min and the supernatant transferred to a 2 mL tube. Solution PPS (250 $\upmu \hbox {L}$) was added to the supernatant and mixed by inversion ten times. The sample was centrifuged again at 14,000 $\times$ g for 5 min and the supernatant transferred to 15 mL tube. The Binding Matrix suspension was resuspended by vortexing for 5 s and 1 mL immediately added to the supernatant, before mixing by inversion for 2 min and settling in a rack for 3 min. Avoiding the Binding Matrix, 500 $\upmu \hbox {L}$ supernatant was removed and discarded and the remaining supernatant and Binding Matrix resuspended by inversion, before 600 $\upmu \hbox {L}$ suspension was transferred to a SPIN filter. The SPIN filter was centrifuged at 14,000 $\times$ g for 1 min and the filtrate discarded. The remaining suspension was added to the SPIN filter and centrifuged again at 14,000 $\times$ g for 1 min. The filtrate was again discarded and 500 $\upmu \hbox {L}$ SEWS-M added to the pellet on the SPIN filter and the pellet gently resuspended using the pipette. The SPIN filter was centrifuged at 14,000 $\times$ g for 1 min, the filtrate discarded and the SPIN filter centrifuged again at 14,000 $\times$ g for 2 min. The catch tube containing the filtrate was discarded and the SPIN filter placed in a clean catch tube and air-dried for 5 min. The pellet on the SPIN filter was gently resuspended in ddH2O (100 $\upmu \hbox {L}$) using a pipette and the SPIN filter incubated for 5 min at $55 {^{\circ }}\hbox {C}$, before a final centrifugation at 14,000 $\times$ g for 1 min. The SPIN filter was discarded and the eluate stored at $-20 {^{\circ }}\hbox {C}$.

For analysis of the microbiome, the V4 region of the small-subunit (SSU) 16S rRNA gene was amplified from genomic DNA using a two-step nested PCR approach (after^84^). Briefly, samples were amplified using primers 519F (5$^{\prime }$-CAG CMG CCG CGG TAA-3$^{\prime }$)^85^ and 806R (5$^{\prime }$-GGA CTA CHV GGG TWT CTA AT-3$^{\prime }$)^86^. The reaction mixture consisted of 10 $\upmu \hbox {L}$ HotStarTaq Master Mix (Qiagen), 500 nM of each primer, 1 $\upmu \hbox {L}$ template DNA, $200\hbox { ng mL}^{-1}$ non-acetylated BSA and nuclease-free water to bring the total volume to 20 $\upmu \hbox {L}$. Reactions were initially denatured for 15 min at $95 {^{\circ }}\hbox {C}$, followed by 25 cycles of denaturation at $95 {^{\circ }}\hbox {C}$ for 20 s, primer annealing at $55 {^{\circ }}\hbox {C}$ for 30 s and extension at $72 {^{\circ }}\hbox {C}$ for 30 s, followed by a final extension step of $72 {^{\circ }}\hbox {C}$ for 7 min. In the second step, pooled amplicons were amplified and barcoded by nested PCR using MID-tagged primers 519F and 806R in a reaction mixture comprising 25 $\upmu \hbox {L}$ HotStarTaq Master Mix, 500 nM of each primer, 1 $\upmu \hbox {L}$ amplicon DNA, and nuclease-free water to bring the total volume to 50 $\upmu \hbox {L}$. Reactions were initially denatured for 15 min at $95 {^{\circ }}\hbox {C}$, followed by 15 cycles of denaturation at $95 {^{\circ }}\hbox {C}$ for 20 s, primer annealing at $62 {^{\circ }}\hbox {C}$ for 30 s and extension at $72 {^{\circ }}\hbox {C}$ for 30 s. This was followed by a final extension step of $72 {^{\circ }}\hbox {C}$ for 7 min. The quantity and quality of the second-step PCR amplicons was assessed by agarose gel electrophoresis. The second-step PCR amplicons were purified using Agencourt AMPure XP Beads (Beckman Coulter Inc., CA, USA) and quantified using a Qubit 3.0 Fluorometer and by agarose gel electrophoresis. MID-tagged amplicons were then pooled in equimolar amounts for library construction. Libraries were sent to the Norwegian Sequencing Centre (Oslo, Norway) for paired-end (2 $\times$ 300bp) HTS on a MiSeq platform (Illumina, CA, USA) using the MiSeq Reagent Kit v3 (Illumina) kit.

### PAC DNA extraction, PCR and sanger sequencing

Tissue (100 mg wwt) was ground with a pestle and mortar and genomic DNA extracted using the DNeasy Plant Mini Kit (Qiagen), as per the manufacturer’s instructions and eluted in a final volume of 200 $\upmu \hbox {L}$.

The phylogenetic identification of the PAC was ascertained by amplification of fragments each of the nuclear-encoded, large-subunit (LSU) ribosomal RNA gene and the *psbA* protein D1 gene (which comprises part of the reaction center of photosystem II) by PCR. Samples were amplified using primers X (5$^{\prime }$-GCA GGA CGG TGG CCA TGG AAG T-3$^{\prime }$) and 28F (5$^{\prime }$-CAG AGC ACT GGG CAG AAA TCA C-3$^{\prime }$)^87^ and primers F40 (5$^{\prime }$-CGT TAT GAG TCA GGT GTA ATT CC-3$^{\prime }$) and R851.1 (5$^{\prime }$-GCC ATT GTT TGA ATA GC-3$^{\prime }$)^88^ for the *LSU* gene and primers psbA-F (5$^{\prime }$-ATG ACT GCT ACT TTA GAA AGA CG-3$^{\prime }$) and psbA-R2 (5$^{\prime }$-TCA TGC ATW ACT TCC ATA CCT A-3$^{\prime }$)^89^ for the *psbA* gene. The reaction mixture comprised 25 $\upmu \hbox {L}$ GoTaq GT HotStart Green Master Mix (Promega), 200 nM of each primer, 1 $\upmu \hbox {L}$ of template DNA (tenfold-diluted in nuclease-free water), 200 ng mL$^{-1}$ non-acetylated BSA and nuclease-free water to bring the total volume to 50 $\upmu \hbox {L}$. Reactions were denatured for 15 min at $95 {^{\circ }}\hbox {C}$, followed by 35 cycles of denaturation at $95 {^{\circ }}\hbox {C}$ for 60 s, primer annealing at 57 and $48 {^{\circ }}\hbox {C}$ for 60 s (for *LSU* and *psbA* genes, respectively) and extension at $72 {^{\circ }}\hbox {C}$ for 2 min, followed by a final extension step of $72 {^{\circ }}\hbox {C}$ for 10 min. The quantity and quality of the PCR amplicons was assessed by agarose gel electrophoresis, prior to excision of bands for sequencing by Genewiz (MD, USA) with the same primers used for PCR.

### Bioinformatic analyses

HTS reads were processed using BBTools (https://jgi.doe.gov/data-and-tools/bbtools/). Briefly: (1) phiX contaminants (calibration controls used during sequencing) were removed; (2) paired end reads were merged; (3) forward and reverse primer sequences and linkers were removed; (4) reads were quality end-trimmed at a phred quality score $\le$ 27; and (5) reads $< 200$ bp in length were removed. The remaining sequence reads were checked for chimeras with the *identify chimeric seqs* and *filter fasta* scripts in QIIME (Quantitative Insights into Microbial Ecology, v1.9.0^90^), using usearch61^91^ and the ChimeraSlayer^92^ 16S rRNA reference database (Gold.fa) found in the Broad Microbiome Utilities suite (http://microbiomeutil.sourceforge.net/). The *pick de novo otus* script in QIIME (using default parameters) was used for de novo OTU picking (using uclust^91^) and a sequence similarity threshold of 97%), taxonomy assignment (using PyNAST^93^) at 90% sequence similarity against the Greengenes core reference alignment database (Release 13_8^94^), and finally, the assembly of a table of OTU abundances with taxonomic identifiers for each OTU.

Sanger FASTQ files were quality end-trimmed at a phred quality score $\le$ 27 using Sickle (Version 1.33)^95^ and processed using EMBOSS^96^. *LSU* and *psbA* sequences were compared with those in the NCBI database using the Nucleotide Basic Local Alignment Search Tool (BLASTn) algorithm^97^.

## Supplementary information

Supplementary Figures.

## Data Availability

HTS data were submitted to the European Nucleotide Archive (ENA) under Study PRJEB39143, whilst PAC marker gene sequences were submitted to ENA under Accession Numbers LR828130-LR828131 (LSU) and LR861111 (psbA).
