# Supplementary material for: An unusual microbiome characterises a spatially-aggressive crustose alga rapidly overgrowing shallow Caribbean reefs
Source: Sci Rep. 2020 Nov 30;10:20949. doi: 10.1038/s41598-020-76204-0 (PMC7705730; doi:10.1038/s41598-020-76204-0)
Supplement: Supplementary file 1 — Supplementary Figures. [file 41598_2020_76204_MOESM1_ESM.pdf]

An unusual microbiome characterises a spatially-aggressive crustose alga rapidly overgrowing shallow Caribbean reefs

Bryan Wilson, Chen-Ming Fan and Peter J. Edmunds

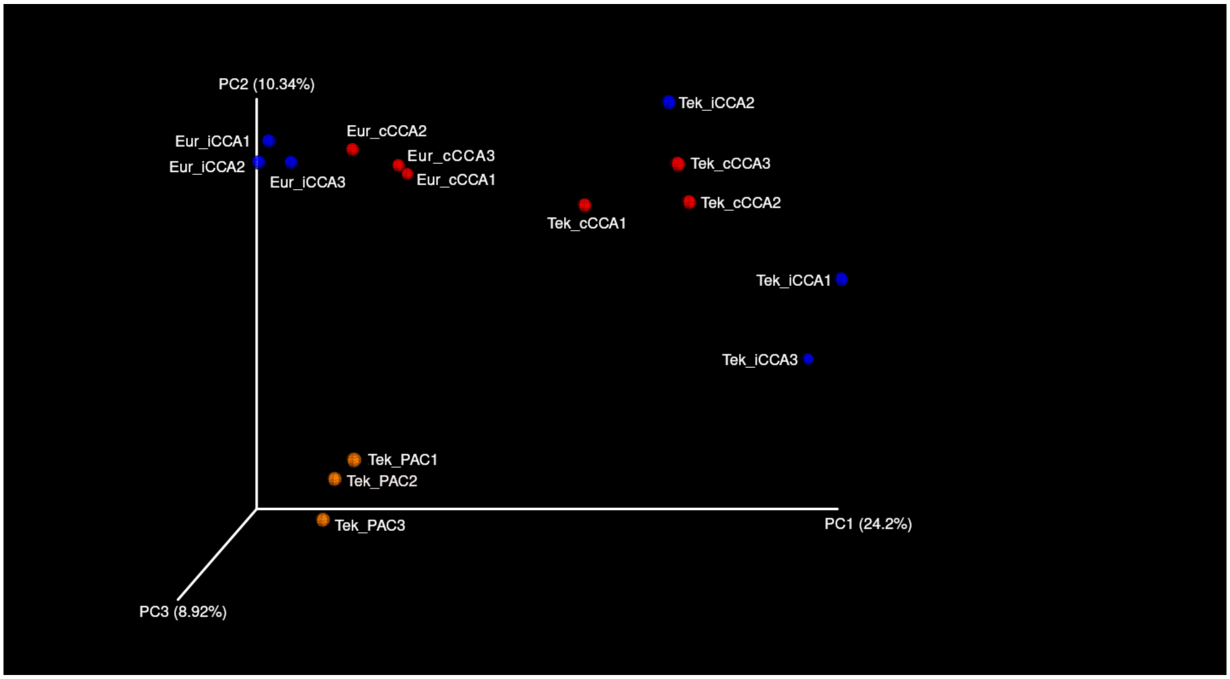

Supplementary Figure S 1. Three-dimensional principle component analyses (PCA) showing the variance in communities of prokaryote OTUs associated with CCA and PAC SM microbiomes sampled from two sites. iCCA = igneous CCA (n=3), cCCA = carbonate CCA (n=3), PAC = peyssonnelid algal crusts (n=3); Sites: Tek = Tektite, Eur = Europa Bay

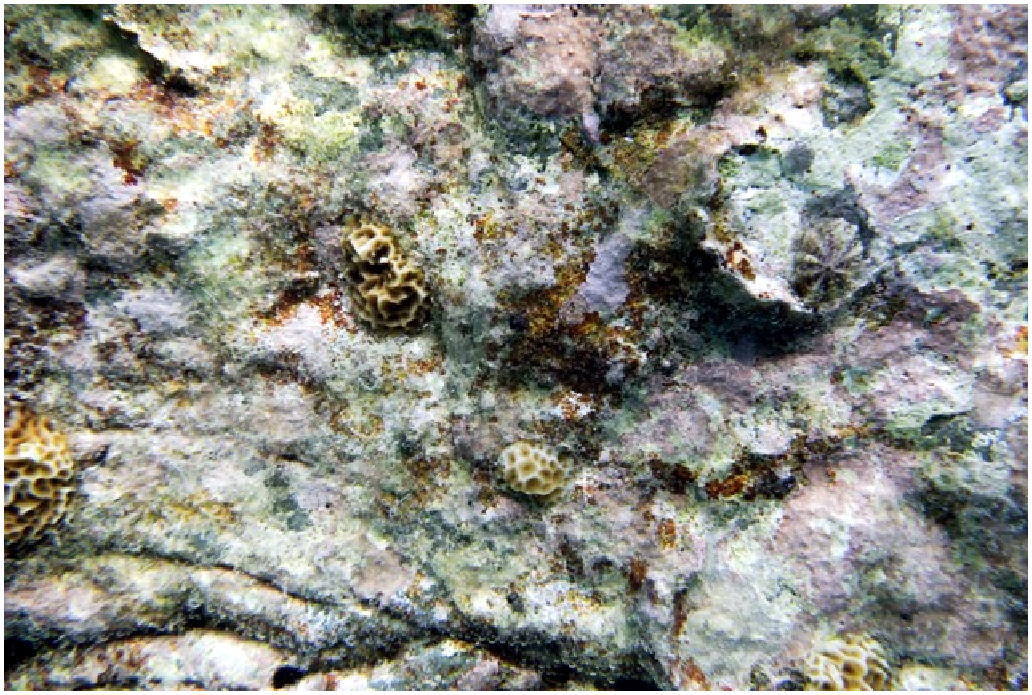

Supplementary Figure S 2. **CCA growing over igneous substrate on reefs in St. John, US Virgin Islands.**

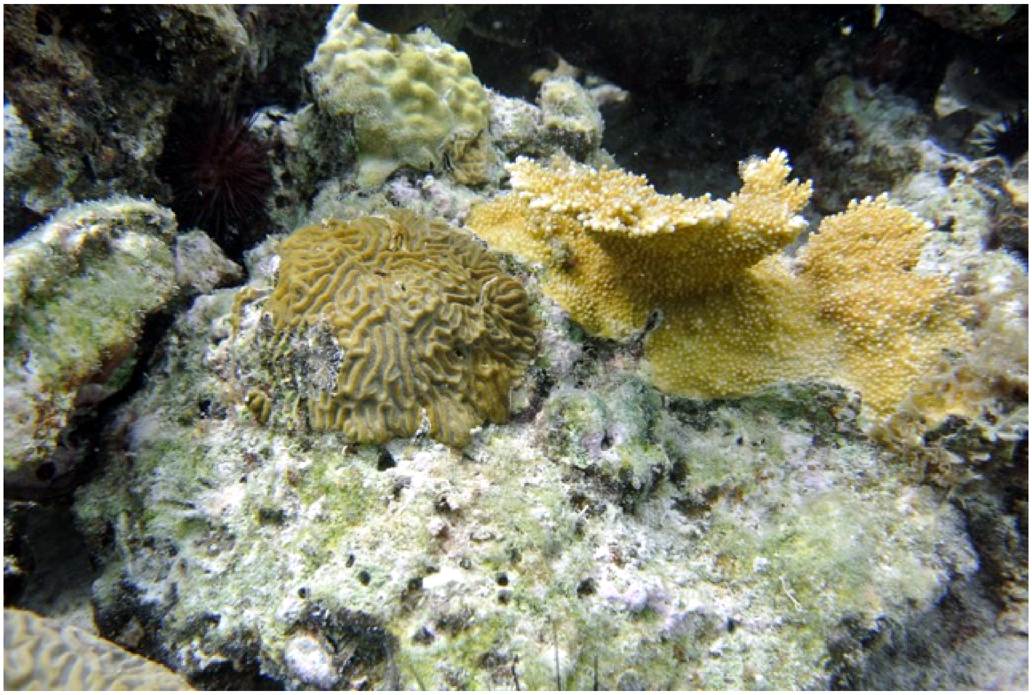

Supplementary Figure S 3. **CCA growing over carbonate substrate on reefs in St. John, US Virgin Islands.**
